# Establishing the behavioral feasibility of a structured story-generation paradigm for neurophysiological research

**DOI:** 10.3389/fpsyg.2026.1920009

**Published:** 2026-09-08

**Authors:** Simrut Kurry, Jasper Delichte, Samantha Yee, Samir Damji, Naznin Virji-Babul

**Affiliations:** 1 Neuroscience Program, University of British Columbia, Vancouver, BC, Canada; 2 Graduate Program in Neuroscience, University of British Columbia, Vancouver, BC, Canada; 3 Department of Physical Therapy, Faculty of Medicine, University of British Columbia, Vancouver, BC, Canada; 4 Djavad Mowafaghian Centre for Brain Health, University of British Columbia, Vancouver, BC, Canada

**Keywords:** creativity, large language model, naturalistic task, story generation task, visual image

## Abstract

Historically, many standard measures evaluate creativity at a single time point and are not suitable for capturing the continuous neural activity underlying naturalistic, creative cognition. The aims of our study were to: (a) establish the feasibility of a behavioral paradigm suitable for investigating the continuous, temporal dynamics of creative cognition using neurophysiological methods, and (b) evaluate the feasibility of using large language models (LLMs) for scoring. Sixty undergraduate students (M age = 21.65 years; M/F = 50%) wrote a brief story integrating three unrelated images into a single narrative. Each story was scored by two large LLMs. The ICC(2,1) for total story scores was 0.67 (95% CI [0.51, 0.79]), indicating moderate agreement between models and were averaged into a composite score. The story task produced a broad distribution of scores (M = 9.2, SD = 2.1; range = 4.5–12.5) supporting its ability to generate variable creative output. We compared the total story creativity score to the Inventory of Creative Activities and Achievements (ICAA) and the Divergent Association Task (DAT). There were no significant correlations with ICAA or DAT scores. A random subset (*n* = 30) of stories rated by two human raters, resulted in moderate agreement with LLM scores (ICC(3,1) = 0.50, 95% CI [0.18, 0.73]). These findings provide preliminary evidence for the behavioral feasibility and characterization of a structured, temporally extended naturalistic, narrative paradigm that can be integrated with neurophysiological methods. Future studies will examine the associated neural signatures and further explore LLM scoring with human raters.

## Introduction

Human creativity is often described as the ability to generate novel and appropriate ideas (Guilford, 1967). It is more broadly understood as the capacity to produce ideas or solutions that are both novel and valuable, emerging from a dynamic interplay of multiple cognitive processes, including memory, attention, executive function, and emotion (Wiggins and Bhattacharya, 2014). Historically, creativity research has relied on two broad classes of assessment: self-report questionnaires and performance-based tasks. Self-report measures, such as the Inventory of Creative Activities and Achievements (ICAA), provide insight into real-world creative behaviors across domains; however, they are inherently subjective, shaped by self-perception and memory, and reflect past rather than current creative engagement. In contrast, performance-based tasks aim to assess creative ability under controlled conditions but typically emphasize brief, decontextualized responses. As a result, these approaches often capture creative potential rather than unfolding processes of creative production, and are difficult to integrate with neurophysiological methods that aim to measure cognition as it dynamically unfolds.

A dominant theoretical framework distinguishes between divergent and convergent thinking processes (Cropley, 2006). Divergent thinking involves the generation of multiple, novel ideas and reflects flexible, expansive cognition, whereas convergent thinking involves narrowing possibilities to identify the most appropriate or effective solution (Cropley, 2006; Pinkow, 2023). Creativity is increasingly understood as emerging from the interaction of large-scale brain networks supporting these processes. However, most existing tasks isolate divergent or convergent thinking and assess them over short time windows, limiting their ability to capture how the underlying neural processes interact dynamically over time (Beaty et al., 2016). Thus there is a need for experimental paradigms that can capture creative cognition as temporally extended process rather than as a series of isolated responses.

A growing body of neurophysiological research has begun to examine the brain dynamics underlying creative cognition, with electroencephalography (EEG) emerging as a particularly informative tool given its temporal resolution. Studies have consistently linked creative ideation to increased alpha-band synchronization (8–12 Hz) in frontal and parietal regions, interpreted as reflecting internally oriented attention and top-down control processes that support idea generation (Benedek et al., 2011; Fink and Benedek, 2014; Jauk et al., 2012). In parallel, large-scale brain networks including the default mode and cognitive control networks, have been shown to differentially contribute to generative and evaluative phases of creative thinking (Beaty et al., 2016; Shi et al., 2018). However, this body of work has relied predominantly on brief, single-trial paradigms—such as the Alternate Uses Task (AUT), Remote Associates Test, and word association tasks, in which creative cognition is compressed into isolated responses lasting only seconds to a minute. While these paradigms have provided important insights into the neural correlates of discrete creative acts of cognitive flexibility, they are less well suited to capturing how neural dynamics evolve across the sustained, multi-phase processes that characterize real-world creative work. Consequently, the neurocognitive mechanisms underlying transitions between phases of creative thinking remain poorly understood. Importantly, such paradigms are needed not primarily as new psychometric tools, but as experimental probes capable of capturing the evolving neural dynamics underlying creative cognition.

One promising approach to address this gap is story generation. Unlike traditional measures, a story-writing task requires individuals to generate novel ideas, characters, and events (divergent thinking) while simultaneously organizing those ideas into a coherent narrative structure (convergent thinking). In this way, it engages multiple components of creative cognition within a single, temporally extended activity. Additionally, narrative output can be evaluated across multiple dimensions, including originality, coherence, and elaboration. Despite this potential, relatively few studies have employed story generation tasks in studying creative behavior (Bauer and Gilpin, 2023; Howard-Jones et al., 2005; Joy and Breed, 2012; Taylor et al., 2021; Wu et al., 2019; Orwig et al., 2024; Doshi and Hauser, 2024). Importantly, such tasks may be well suited for integration with neurophysiological methods, as they involve continuous cognitive engagement within a controlled yet flexible framework.

A key challenge in using open-ended narrative tasks, however, lies in the evaluation of creative output. Traditional approaches rely on human raters, which are time-intensive and difficult to scale, particularly for complex, multi-dimensional responses. Recent advances in large language models (LLMs) offer a potential alternative. AI-based methods have been successfully applied to the assessment of creativity in divergent thinking tasks (see Luchini et al., 2025a,b for review), and emerging evidence suggests that LLMs can provide structured and scalableevaluations of creative outputs under certain conditions, particularly when guided by structured prompts or examples (e.g., Luchini et al., 2025a,b). More recently, Primi et al. (2026) demonstrated that AI-based scoring systems can achieve substantial agreement with human ratings across languages and task formats. Together, these recent developments provide emerging evidence for a scalable and standardized framework for evaluating complex, naturalistic creative outputs, making them particularly well suited for studies that aim to integrate behavioral and neurophysiological data.

The present study introduces and pilots a novel, structured, story generation task designed to elicit creative production within a constrained yet flexible narrative context. Participants were required to generate a short, written story incorporating multiple unrelated visual images, encouraging both idea generation and narrative organization over time. Importantly, the present paradigm was not intended to replace established creativity tasks such as the AUT or DAT which have demonstrated utility for both behavioral and neurophysiological research. The goals of the present report were to: (a) establish the feasibility of a behavioral paradigm suitable for investigating the continuous, temporal dynamics of creative cognition using neurophysiological methods, and (b) evaluate the feasibility of using large language models (LLMs) ratings as a scalable approach for evaluating narrative outputs. Importantly, the behavioral output is not proposed as a standalone psychometric measure of individual creativity; rather, it provides a naturalistic, cognitively demanding context in which to examine transitions from rest into sustained creative cognition involving coordinated divergent and convergent processes. This approach therefore complements more constrained creativity tasks by enabling the investigation of temporally extended brain dynamics.

## Methods

### Participants

Undergraduate students were recruited through flyers and campus-based advertisements and were reimbursed for their time and travel. Inclusion criteria included being undergraduate students between the ages of 19–25 years, right handedness, fluency in English to ensure comprehension of task instructions, and no ongoing neurological illnesses, injuries, or conditions.

The final sample included 60 participants (M age = 21.65 years; %M/F = 50%). Academic backgrounds were varied, with participants majoring in social sciences, natural sciences, health sciences, engineering and applied sciences, and business. All participants completed all study measures. A target sample of 60 participants was selected for this feasibility study to assess task completion, adherence to task constraints, variability in narrative responses, and the practicality of the scoring approach rather than to provide sufficient power for psychometric validation.

Thirty participants from the present sample also participated in a companion study examining *resting-state* EEG network profiles associated with creativity-related individual differences. Participants in that study completed a standardized creativity assessment battery including the ICAA and DAT, during a separate session in which resting-state EEG was recorded prior to task engagement. The present study and that work were designed as complementary components.

To protect privacy, all data collected was anonymized and stored under non-identifiable participant IDs. The study was approved by the University of British Columbia Behavioural Research Ethics Board (H22-01386) in accordance with the Helsinki declaration. All research was performed in accordance with relevant guidelines/regulations. All participants provided informed consent prior to participation. All participants received financial compensation for their travel and parking after completion of the study.

#### Study design and procedure

Participants first completed an online Qualtrics survey, which included a consent form, demographics questionnaire, a self-reported creativity rating on a 7-point Likert scale and an established self-report questionnaire: the Inventory of Creative Activities and Achievements (ICAA) (Diedrich et al., 2018), scored according to established guidelines. During the in-person study session, participants first completed an established performance-based task, the Divergent Association Test (DAT) (Olson et al., 2021), administered via datcreativity.com, in which participants generated 10 semantically unrelated nouns within 4 min. Responses were scored using the platform’s automated algorithm. The ICAA and DAT measures were included to examine the extent to which the story task scores correlated with established approaches to creativity assessment.

This was followed by the story generation task to capture naturalistic creative writing. This task was designed to elicit creative production within a structured, time-constrained format. Participants were instructed to write a short story in response to the following prompt: “You have a big paper worth 70% of your grade due tomorrow. You have not started and will not get it done on time. Write an excuse letter to your professor explaining why you were unable to get this done. You will be shown three separate images that you should incorporate into your story in a specified order: the first image at the beginning, the second in the middle and the third at the end”.

**Figure 1:**
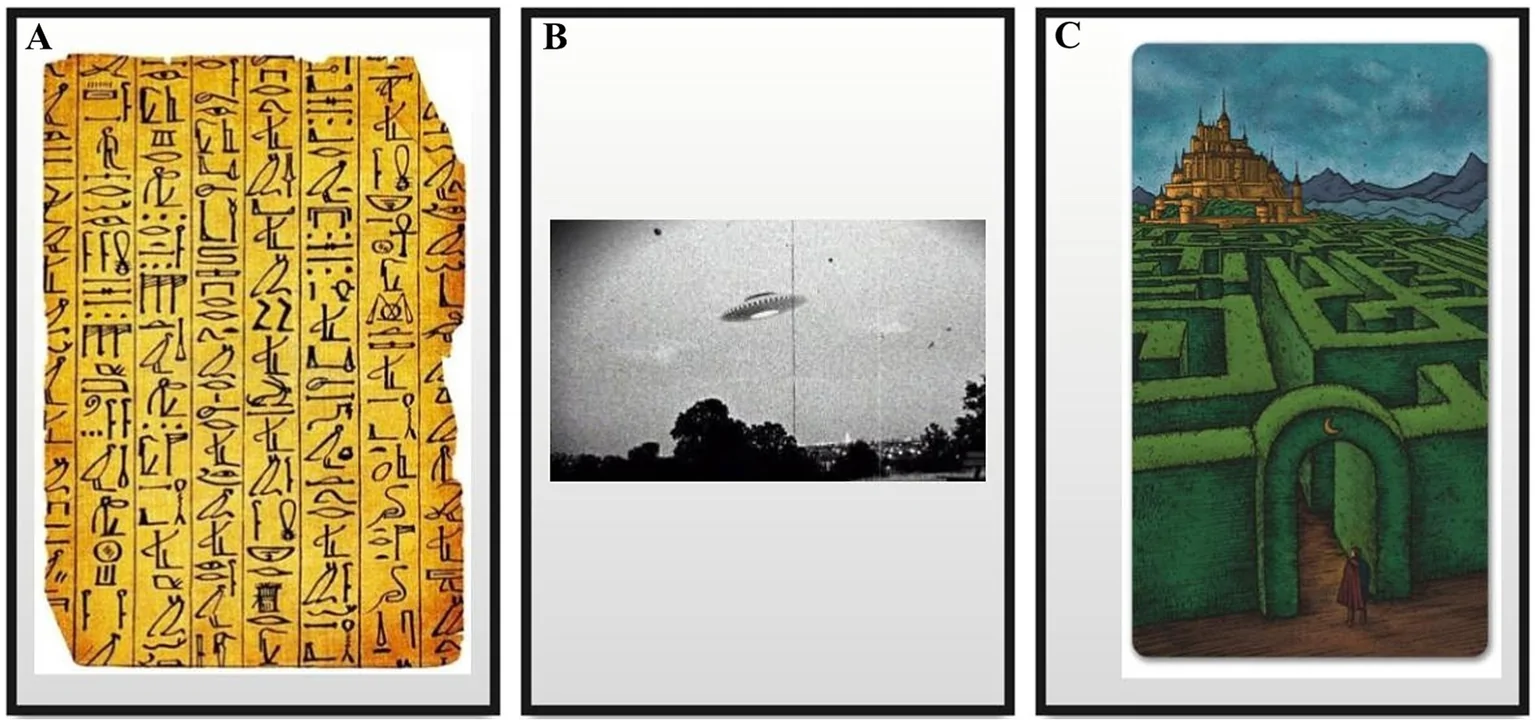
Visual stimuli used in the story generation task. Participants were instructed to incorporate the three images in the specified sequence: **(A)** hieroglyphics, **(B)** spaceship, and **(C)** maze.

All participants received the same images in the same sequence as shown in Figure 1. The images remained visible throughout the task. The task consisted of a one-minute planning phase during which participants were instructed not to type, followed by a five-minute writing phase during which participants typed their story into a blank document. A timer was visible throughout the task.

The prompt was selected to be familiar and broadly accessible while introducing a constraint that required creative justification with pre-selected unique images. These images were selected to be non-academic and atypical to discourage stereotyped responses (Howard-Jones et al., 2005) and were piloted to ensure suitability for the task objectives. Feasibility was operationalized as (a) task completion, (b) adherence to task constraints, and (c) variability in response outputs.

#### LLM based story evaluation

Two open-access LLMs were selected to provide a and consistent method for quantifying narrative output, enabling future integration of behavioral and neurophysiological data. Specifically, we used OpenAI ChatGPT 5.2 and Anthropic Claude 3.5 Sonnet using web-based interfaces. Each model independently rated all stories on three dimensions: originality, coherence, and elaboration. These dimensions were adapted from Urban et al. (2024) and Todd et al. (2019). Each dimension was scored on a 5-point scale (1 = very low to 5 = very high). All stories were evaluated in one session. The structured prompts used to guide the models are presented in Supplementary Box 1.

Each model was first provided with a practice example to ensure comprehension of the task. An initial subset of five stories was scored to confirm procedural consistency. No modifications to prompts or scoring procedures were made following this step. All subsequent stories were evaluated using identical instructions. For each story, dimension scores were extracted and averaged across models to reduce model-specific bias. A composite score summing all three dimensions was then produced for each story, ranging from a possible minimum score of 3 to a possible maximum score of 15. *Human Rater Benchmark (Exploratory).*

To provide an exploratory comparison with human judgment, two trained human raters independently scored a random subset of 30 of the 60 stories (50%) using the same rubric and 5-point scale as the LLMs. Each story was evaluated on originality, coherence, and elaboration, with a composite score calculated by summing the three dimension scores. Raters were blind to each other’s ratings and to the LLM-derived scores.

#### Data analysis

Descriptive statistics were computed to characterize performance on the story generation task, including variability in total and dimension scores. Task success was operationalized in terms of variability in responses and task completion across participants. Inter-rater reliability between models and between models and human raters was assessed using intraclass correlation coefficients (ICC). Agreement between LLM scores and human ratings (*n* = 30) was examined as an exploratory benchmark using ICC. Pearson’s correlations were conducted to examine relationships between story generation scores and other creativity measures (DAT, ICAA, and self-report creativity). Primary outcome variables included story-based total creativity scores and scores from established creativity measures.

## Results

### Story generation task

All participants successfully completed the story generation task, indicating high task compliance. In addition, all participants incorporated the required images in the specified sequence. Table 1 shows a sample of 3 stories.

**Table 1:** Sample of three stories illustrating a broad range of narrative content.

**Story A** Dear Dr. X, I am writing to you today regarding my term paper. I had thought that the best way to immerse myself in the field of Ancient Egypt was to actually travel there to study the hieroglyphics. So that is where I went during my reading break. However, while I was leaving the pyramids, I noticed the most unique combination of symbols that warned of imminent danger. I knew I had to stay and help. As I went back to my hotel to alert my local guide, I saw a CFO flying above the ground. It abducted me. We traveled for hours and hours, until finally, with no warning, it dropped me into a maze and told me that my goal was to save Prince Charming, who was being held captive in his palace by a dragon. I had to navigate my way through the maze. It took a while, but I got through to him and befriended the dragon, who has now basically become my chauffer. All of this is to say that, as the new princess, my term paper will be a little bit late, as now I have all of these tasks to help me prepare to become the queen. I look forward to your response, and to submitting as soon as possible. Best, Princess XXX
**Story B** Dear Professor, I am writing to you to ask for an extension on my final assignment worth 70% of my final grade. Unfortunately my laptop was destroyed yesterday because of an engineering project I was working on. My team and I had to decode a code that was given to us to be able to build a functional spaceship as a class project. We did not properly decode it and the ship exploded along with my laptop and few other members’ belongings. My laptop had the final copy of my assignment and I tried to print it before getting to my spaceship engineering project, but there was a carnival that settled in front of the store and they built a huge maze that was blocking the path to the store. Therefore, I did not have enough time to print my paper before needing to get to my engineering class. I was planning on going after but my laptop exploded.
**Story C:** While I was walking home from school yesterday to finish my paper, I was kidnapped by a gang of Russian mobsters who jumped out of a van and put a bag over my head. I was gagged and bound and brought to a discreet warehouse outside the city. When they finally took the bag off my head, I found myself in a room covered in unfamiliar symbols which seemed like Egyptian hieroglyphics. They yelled at me in Russian, which I did not understand, but when I finally said, “I can bring you what you want,” they let me go and I bounced out of the chair they had tied me in. I walked out to point out the alien landing site which had just come around last week. I told the mobsters the aliens would give them the weapons they wanted. So the gangsters and I began walking over to the aliens, but before we could get there, we found a giant labyrinth in front of the alien spacecrafts. There were some instructions in front of the maze, but they were in the same strange symbols I saw in the gang warehouse. Disregarding the instructions, the Russians moved through the maze. However, we seemed to move in circles the entire time without going anywhere. After an hour, the aliens descended upon us and turned all the gangsters into rats. I escaped with my life, but it was already midnight.

Descriptive statistics for the story task and additional creativity measures are presented in Table 2. The total story score had a mean of 9.20 (SD = 2.10), with scores ranging from 4.50 to 12.50. This distribution suggests that the task elicited a range of responses without evidence of floor or ceiling effects, consistent with its use in capturing variability in narrative output within a structured format. Descriptive statistics for the ICAA, Divergent Asssociation Test (DAT), and self-reported creativity are included in Table 2 to provide context relative to established measures.

**Table 2:** Descriptive statistics of scores across creativity measures.

Behavioral measure	Mean	SD	Min	Max	Published comparison values
ICAA creative total	162.21	59.94	39	360	
ICAA creative activity	74.04	24.91	24	131	0.00–124 (Jauk et al., 2014) Mean: 54.51 (Diedrich et al., 2018)
ICAA creative achievement	88	41.47	8	231	0.00–208 (Jauk et al., 2014) Mean: 113.9 in arts students (Diedrich et al., 2018)
Divergent association task	82.13	6.02	66.24	95.74	Range from 75 to 80 few exceeding 100 (Olson et al., 2021)
Self-reported score	4.48	1.06	2	7	Mean: 4.38 (Oh and Pyo, 2023)
Story task total	9.2	2.1	4.5	12.5	

### LLM-based scoring

Inter-rater reliability between the two large language models was assessed using an intraclass correlation coefficient (ICC). The ICC(2,1) for total story scores was 0.67 (95% CI [0.51, 0.79]), indicating moderate agreement between models. This level of agreement is consistent with the use of LLMs as a potential approach for generating consistent evaluations of open-ended narrative responses, while also indicating that ratings are not fully interchangeable across models. Following this analysis, scores from the two models were averaged to create a composite creativity score for each story, which was used in subsequent analyses.

### Human rater benchmark (exploratory)

Consistency between the LLM ratings (Claude and ChatGPT) and mean human ratings was moderate for the composite score (ICC(3,1) = 0.50, 95% CI [0.18, 0.73]) and varied across dimension, ranging from 0.41 for coherence to 0.56 for elaboration and originality at 0.49. These findings provide a preliminary comparison between LLM and human ratings and should be interpreted cautiously.

### Exploratory analyses

Pearson’s correlations were conducted to examine associations between story-based creativity scores and other measures of creativity (self-report, ICAA, and DAT). No significant correlations were observed after correction for multiple comparisons. A small positive association between story scores and self-reported creativity was observed (*r* = 0.25, *p* = 0.050), but this did not remain significant after correction. Correlations with ICAA total scores (*r* = −0.03, *p* = 0.822) and DAT scores (*r* = 0.17, *p* = 0.202) were not significant. Results are presented in Table 3.

**Table 3:** Correlations between scores across creativity measures.

Measure	*r*	*p* (uncorrected)	*p* (Bonferroni)	*p* (FDR)
Self-reported creativity	0.254	0.050	0.150	0.150
ICAA_total	−0.030	0.822	1.000	0.822
DAT	0.167	0.202	0.607	0.304

## Discussion

The present study evaluated the feasibility of a structured story generation task as an experimental paradigm for eliciting temporally extended creative cognition suitable for neurophysiological investigation, along with the feasibility of using LLMs to efficiently quantify the narrative outputs. Overall, our findings provide preliminary evidence that the story generation task can generate variable and scorable narrative responses under controlled conditions.

The story generation task was successfully completed by all participants and produced substantial variability in scores, indicating that it can effectively elicit differentiated narrative outputs within a constrained format. Importantly, the task was designed to engage both idea generation and organization processes within a single activity. The observed variability, alongside the absence of ceiling or floor effects, suggests that the task is sensitive to individual differences while maintaining sufficient structure for experimental use. However, the extent to which the task captures specific components of creative cognition cannot be determined from the present data alone. We wish to be explicit that this study was not designed or intended as a validation of a new psychometric creativity instrument; accordingly, the absence of significant correlations with the ICAA and DAT (reported below) is not interpreted here as evidence against the task’s value, but rather reflects that convergent validation against established measures was outside the present scope. We also note that, because the task requires generating and organizing narrative content, performance likely draws on narrative and language proficiency in addition to creative ideation, a possibility we discuss further in the Limitations.

The second aim was to evaluate the feasibility of LLM-based scoring. Inter-rater reliability between the two models was moderate (ICC = 0.67). This level of agreement suggests that LLM-based scoring may offer a potentially useful approach for evaluating open-ended responses, particularly when scalability is required. At the same time, the moderate agreement suggests that model outputs are not interchangeable and may reflect differences in training data or evaluative criteria. While emerging literature is showing that LLMs can approximate human judgments of creativity under structured conditions (DiStefano et al., 2025; Luchini et al., 2025a,b; Organisciak et al., 2023), they also highlight that automated scoring remains an evolving methodological area (Benedek and Beaty, 2025; Saretzki and Benedek, 2026). In the present context, agreement between two LLMs does not establish validity relative to human judgments. The present analysis only provides evidence of consistency between two models and a behavioral index for the present paradigm.

Exploratory analyses did not reveal significant associations between story-based creativity scores and established measures (ICAA and DAT), and only a small, non-significant association with self-reported creativity. These findings should therefore be interpreted cautiously. The lack of significant correlations may reflect limited statistical power, differences in task format, or partially overlapping constructs. The ICAA captures accumulated real-world creative engagement, whereas the DAT assesses a specific aspect of divergent thinking under constrained conditions. In contrast, the story generation task requires the interplay of multiple cognitive processes within a sustained and context-dependent format. The lack of strong convergence across measures is therefore consistent with theoretical accounts emphasizing the multidimensional and context-dependent nature of creativity (Cropley, 2006; Eymann et al., 2026) and the need to bridge established measures with tasks involving naturalistic, cognitive demands.

## Limitations

Several limitations should be noted. First, the sample was restricted to undergraduate students at a single university, which may limit generalizability. Second, the relatively small sample size (N = 60) reduced statistical power and may have precluded the detection of small but meaningful associations between measures. Third, although LLM-based scoring showed moderate reliability and offers practical advantages related to resources, the present validation against independent human raters was exploratory and based on a modest subsample (*n* = 30). More extensive validation involving larger samples remains an important direction for future work to establish the robustness and generalizability of LLM-based scoring for this paradigm. Fourth, because this task is inherently language-based, performance likely reflects verbal and narrative proficiency in addition to creative ideation. This confound is shared with related association-based tasks (e.g., the Alternate Uses Task) and future iterations will include a measure of verbal fluency as a covariate.

## Future directions

The central motivation of this work was to establish a structured narrative paradigm to interrogate the temporal dynamics of creative cognition in a controlled yet naturalistic setting. This methodology will serve as an important tool connecting resting state and task-based EEG signatures to behavioral measures of creativity. Work already underway in our laboratory has begun to address this by exploring resting-state EEG network profiles associated with individual differences in established measures in a related sample. The present report complements and extends this work by establishing a paradigm to capture creative cognition as it unfolds, enabling the examination of task-engaged neural dynamics that resting state cannot access. The present findings also demonstrate that the task reliably elicits sustained, variable, creative output within a format that is compatible with experimental constraints, providing a foundation for its use in neurophysiological research.

The next step is to integrate this paradigm with EEG to study the evolving neural processes underlying creative cognition as they unfold over time. EEG offers the temporal resolution necessary to resolve rapid transitions between cognitive states, making it particularly well suited to studying extended forms of creative production. The design of the task, with distinct planning and writing phases embedded within a continuous narrative context provides a tractable framework for examining how neural activity shifts between generative and organizational processes. By integrating the present paradigm with EEG, we plan to examine how the intrinsic network signatures are dynamically recruited and reorganized during active narrative construction. Oscillatory dynamics associated with internally directed attention, cognitive control, and associative processing can be examined in relation to specific phases of narrative construction, enabling a more fine-grained account of how creative cognition emerges over time.

Concretely, we envision segmenting the EEG recording into three task-defined epochs aligned to the behavioral paradigm: a pre-task baseline, the one-minute planning phase, and the five-minute writing phase (further subdivided into early, middle, and late thirds to capture the beginning-middle-end narrative structure imposed by the image sequence), Within these epochs, we plan to analyze graph-theoretical connectivity indices linking default-mode and executive control network engagement, consistent with markers established in prior single-trial creativity paradigms (Benedek et al., 2011; Beaty et al., 2016). These epoch-specific neural indices would then be related to the behavioral composite and scores described here, providing a concrete, testable link between the temporal structure of narrative construction and the evolving neural signatures that motivate this paradigm. We view the present behavioral report as the first stage of this two-stage research program: establishing the feasibility, variability, and scorability of the task itself with EEG data collection and this analytical framework to be reported in a subsequent study.

This approach to connecting behavioral and neural signatures related to creativity builds on prior work demonstrating the utility of EEG for characterizing neural dynamics during resting state in both healthy controls and in individuals experiencing cognitive symptoms of brain fog resulting from brain injury (Sattari et al., 2026) and the side effects of chemotherapy (Damji et al., 2025a,b). By combining a temporally extended behavioral paradigm with high-resolution neural measurement, this framework is positioned to move beyond static or single-response tasks toward a more dynamic account of creative cognition as it unfolds in real time.

## Conclusion

The present study establishes a feasible behavioral paradigm that produces sustained, variable narrative output suitable for future integration with neurophysiological methods. The task is not proposed as a validated psychometric measure of individual creativity, nor does it function as a standalone assessment tool. It’s primary contribution lies in supporting the integration of behavioral and neurophysiological data within more holistic experimental contexts.

The use of LLM-based rubric scoring provides a practical and consistent means of quantifying complex narrative outputs, although the moderate agreement observed with human raters indicates this approach requires further validation before broader adoption. Within this framework, examining how brain dynamics evolve during cognitive activity or are perturbed from a baseline resting state serves as a novel neural probe for understanding behavioral variability in the creative literature. Taken together, these findings position structured narrative generation as a promising approach for capturing the unfolding dynamics of creativity, opening the door to investigations that resolve how creative cognition is organized over time and across contexts at the neural level.

## Data Availability

The raw data supporting the conclusions of this article will be made available by the authors, without undue reservation.
