# Supplementary material for: Establishing the behavioral feasibility of a structured story-generation paradigm for neurophysiological research
Source: Front Psychol. 2026 Sep 8;17:1920009. doi: 10.3389/fpsyg.2026.1920009 (PMC13593885; doi:10.3389/fpsyg.2026.1920009)
Supplement: Supplementary file 1 [file Data_Sheet_1.PDF]

Supplementary Box 1: Structured prompts used to guide the LLM models.

Prompt 1: *Your task is to evaluate the creativity of short stories that were written by undergraduate students in response to the following prompt:*

"You have a big paper worth 70% of your grade due tomorrow. You have not started and will not get it done on time. Write an excuse letter to your professor explaining why you were unable to get this done. You must incorporate the following images into your story (hieroglyphics, spaceship and maze) in a specified order: the first image at the beginning, the second in the middle, and the third at the end".

Your task is to evaluate the story along three dimensions:

A. *Originality: How novel or creative are the ideas? Does the story include unexpected or imaginative elements?*
B. *Coherence: Is the story logically organized? Does it flow clearly from beginning to end?*
C. *Elaboration: How detailed is the story? Are ideas developed with sufficient depth?*

Use the following rating scale for each dimension: 1 = Very low; 2 = Low; 3 = Moderate; 4 = High; 5 = Very high

Prompt 2: *Do you understand the task? Let's start with a practice story. Read the story and evaluate using the three dimensions.*

Prompt 3: *Read all the 60 uploaded stories. Create an Excel sheet with columns for the participant ID and each of the three dimensions. Score the first 5 stories only. Provide a score (1-5) for each dimension and enter the scores on the Excel sheet. Briefly justify the rationale for your score. Base your ratings only on the content of the story. Do not infer intent beyond what is written; Use the full range of the scale; Be consistent across evaluations*

Prompt 4: Read the remaining 55 uploaded stories. Score (1-5) for each dimension, briefly justify the rationale for your score. Base your ratings only on the content of the story. Do not infer intent beyond what is written; Use the full range of the scale; Be consistent across evaluations. Enter the scores on the same Excel sheet.
